# Infection prevention and control procedures in nursing homes in the piedmont region of Italy: results from two regional surveys (2021–2024)

**DOI:** 10.1186/s13756-026-01796-7

**Published:** 2026-07-25

**Authors:** Valerio Bordino, Vittoria Nunziata, Anna Laura Busca, Giuseppina Dassio, Daniela Granatiero, Lorenza Ferrara, Bartolomeo Griglio, Carla M. Zotti, Costanza Vicentini

**Affiliations:** 1 Dipartimento di Prevenzione, ASL TO5, Moncalieri, Italy; 2 Department of Public Health and Pediatrics, University of Turin, Turin, Italy; 3 Settore Residenzialità, ASL Città di Torino, Turin, Italy; 4 DIRMEI: Dipartimento Interaziendale Funzionale A Valenza Regionale “Malattie Ed Emergenze Infettive”, Turin, Italy; 5 Regione Piemonte - Direzione Sanità- Prevenzione, Sanità Pubblica Veterinaria e Sicurezza Alimentare, Turin, Italy

## Abstract

**Background:**

Infection prevention and control (IPC) in long-term care facilities (LTCFs) is essential to reduce the burden of healthcare-associated infections (HAI) and the spread of antimicrobial resistance (AMR). This paper describes the development of standardized IPC procedures through a stakeholder-informed approach, and integrates results from two regional surveys conducted in Piedmont (first survey 2021–2022; second survey 2024) which assessed the reported availability of written procedure documents in nursing homes and, subsequently, the uptake and perceived utility of the developed standardized procedures.

**Methods:**

Two anonymous cross-sectional surveys targeted all nursing homes registered in the Piedmont regional healthcare census. The first survey (December 2021-April 2022) assessed the reported inclusion of 16 IPC procedure domains in facility manuals and the date of last revision. Based on these findings, standardized IPC procedure documents were developed by the University of Turin in collaboration with DIRMEI, regional public health authorities and IPC stakeholders, and were made available online in 2023. A second survey (August 2024) assessed reported uptake, perceived adequacy, missing but useful procedures and overall satisfaction. Descriptive statistics were used.

**Results:**

436/780 facilities responded to the first survey (55.9%). High availability of written procedure documents was reported for hand hygiene (99.1%), isolation for COVID-19 (99.8%) and cleaning/sanitation (98.9%). Lower availability was reported for sterilization (46.2%), bloodstream infection prevention (45.9%), and management of colonized/infected patients for alert organisms (62.0%). Revision dates varied, with many critical procedures updated predominantly in 2020–2022. After dissemination of standardized procedures, 305 facilities (39.1%) participated in the second survey: 99.7% found them adequate, 31.1% reported possessing all procedures already, and 89% provided an overall positive judgment. The most frequently requested/adopted procedures were vaccination prophylaxis (33.8%), laboratory sample management (31.8%), TB control (26.2%) and catheter management (22.0%).

**Conclusions:**

This regional initiative identified heterogeneity in IPC procedural documentation in nursing homes and documented high perceived adequacy and usefulness of standardized procedure documents after dissemination. These findings support harmonized regional resources as a structural component of IPC governance in LTCFs. However, the surveys did not assess implementation fidelity, staff adherence, audits, HAI rates, antimicrobial use or AMR outcomes; future work should incorporate objective implementation and outcome indicators.

## Introduction

Healthcare-associated infections and the escalating crisis of antimicrobial resistance collectively represent increasingly significant and complex threats to global public health. [1, 2] These profound challenges are especially salient within long-term care facilities (LTCFs), including nursing homes, given their highly vulnerable population. Residents in these institutional settings face an inherently elevated risk profile, stemming from multiple factors such as advanced age, chronic multimorbidity, and the shared nature of living environments, all of which unfortunately accelerate the transmission of pathogenic microorganisms. [3, 4] The COVID-19 pandemic further highlighted the vulnerability of nursing homes and the importance of clear, updated and context-appropriate infection prevention and control (IPC) procedures. [5–9].

In Italy, national policy frameworks such as the National Plan for Combating Antimicrobial Resistance (PNCAR) and the National Prevention Plan (PNP) emphasize IPC, AMR containment and organizational preparedness across healthcare and territorial care settings. [10, 11] In Piedmont, regional coordination on HAI and AMR prevention has been supported by the GLICA technical group and by regional public health structures. However, at baseline there was no single harmonized set of IPC procedure documents specifically designed for all nursing homes in the region, as the legal requirement does not mandate a specific set of procedures, but rather requires demonstrating attention to IPC by implementing relevant measures. Facilities therefore relied on locally developed or adapted documents, with potential variability in content, updating and operational alignment.

The scientific and public health gap addressed by this study is the limited evidence on how regional health systems can map IPC procedural-document availability in nursing homes and use these findings to develop shared, standardized resources for LTCF settings. This paper reports original findings from two regional cross-sectional surveys. The first survey mapped the reported inclusion of IPC procedure documents in facility manuals and their revision dates. The subsequent regional initiative developed and disseminated standardized procedure documents. The second survey assessed reported uptake/access, perceived adequacy and perceived usefulness of these documents. We do not evaluate clinical effectiveness or staff adherence; rather, we describe a structural and organizational component of IPC governance in nursing homes.

## Methods

### Study design and setting

We conducted a descriptive study integrating two anonymous cross-sectional surveys and the development/dissemination of standardized IPC procedure documents for nursing homes in Piedmont, north-western Italy. The two surveys were designed to inform and subsequently describe a regional IPC harmonization initiative. They were not designed as a longitudinal effectiveness evaluation.

### Eligibility and participants

All nursing homes registered in the Piedmont regional healthcare census and operating at the time of the surveys were invited to participate. Only facilities providing residential long-term care for older/dependent adults were eligible. Facility managers or designated IPC/quality referents were invited to complete the questionnaire on behalf of the facility. Individual-level resident or staff data were not collected.

### Development and dissemination of standardized procedure documents

Findings from the first survey were reviewed by the University of Turin, DIRMEI, regional public health authorities, and Infection Risk Units of the regional trusts. Existing IPC procedures used in hospitals and healthcare facilities were collected, compared and harmonized. The procedure documents were standardized in structure and formatting and reviewed for consistency with national/regional IPC and AMR guidance. The documents were intended as regionally shared operational resources to be adapted locally where needed, rather than as evidence of direct compliance in daily practice. The final set of procedures was made publicly available online in 2023. [12]

### First survey

The first survey (December 2021–April 2022) used a REDCap-based platform to assess the reported inclusion of 16 IPC procedure domains in facility manuals/procedural documentation and the date of last revision. Domains included COVID-19 isolation procedures, dedicated pathways, management of colonized/infected patients for alert organisms, hand hygiene, prevention of urinary tract infections, prevention of pneumonia/oxygen therapy management, prevention of bloodstream infections/CVC-PVC management, laboratory sample management, TB risk assessment, prevention of occupational accidents and use of PPE, vaccination prophylaxis among healthcare workers, bedding management, cleaning/sanitation, disinfection, sterilization and waste management.

### Second survey

The second survey (August 2024) assessed reported uptake and perceived utility after dissemination of the standardized procedure documents. The questionnaire included five items: (1) whether the facility had accessed or used the standardized procedures; (2) whether the procedures were considered adequate; (3) which procedures were already available/adopted locally; (4) which procedures were missing but considered useful; and (5) an overall 1–3 satisfaction score (1 = negative; 2 = needs improvement; 3 = positive), with open -ended field for comments.

### Data analysis

Responses were summarized using counts and percentages. Percentages in procedure-specific analyses were calculated using the number of valid responses for each item as the denominator; item-level denominators therefore vary. Missing item responses were not imputed. Because the surveys were anonymous and facility-level identifiers were not linked across surveys, direct longitudinal facility-level comparisons were not performed. No inferential tests were used, and no effectiveness, adherence, audit, HAI, antimicrobial use or AMR outcomes were assessed. Analyses were conducted using IBM SPSS Statistics v.28.0.

### Ethical considerations

Ethical approval was not required for this study, as the survey collected only facility-level organizational information and did not include patient/resident identifiers or individual-level clinical data. Participation was voluntary, data were analyzed anonymously, and findings are reported in aggregate form.

## Results

### First survey

Of the 780 invited facilities, 436 responded (55, 9% response rate). Table 1 reports item-level denominators and the proportion of facilities reporting that each procedure was included in their IPC manual or procedural documentation. Denominators vary because not all respondents answered each procedure-specific item.

**Table 1:** Reported availability, missing status, and historical revision timelines of infection prevention and control (IPC) procedures across surveyed facilities (N = 436)

Procedure	Total responses	Procedure document reported as included: n (%)	Procedure document not reported as included/missing	Total number of reported revision dates: n (%)	Oldest revision date	Latest revision date	Average
Isolation procedures, with a main focus on COVID-19	435	434 (99.77%)	1	421 (97.00%)	01/03/2020	04/03/2022	04/09/2021
Hand hygiene for healthcare workers	434	430 (99.08%)	4	412 (95.81%)	09/02/2009	04/04/2022	19/01/2021
Cleaning/sanitation	433	428 (98.85%)	5	407 (95.09%)	08/04/2008	03/04/2022	18/09/2020
Preparation of dedicated pathways	436	419 (96.10%)	17	405 (96.66%)	01/03/2020	29/03/2022	04/08/2021
Waste management	433	413 (95.38%)	20	388 (93.95%)	15/05/2006	01/09/2021	29/05/2020
Prevention of occupational accidents and use of personal protective equipment (PPE)	432	406 (93.98%)	26	386 (95.07%)	01/01/2014	01/04/2022	19/01/2021
Disinfection	430	398 (92.56%)	32	372 (93.47%)	01/09/2006	10/11/2021	06/09/2020
Bedding management for staff and guests	433	400 (92.38%)	33	378 (94.50%)	30/06/2006	14/02/2022	12/07/2020
Prevention of vaccine preventable diseases: vaccination prophylaxis in healthcare workers (main focus on COVID)	424	294 (69.34%)	130	273 (92.86%)	01/01/2019	22/04/2022	05/07/2021
Prevention of urinary tract infections- insertion and management of urinary catheterization	430	284 (66.05%)	146	261 (91.90%)	01/01/2008	01/04/2022	15/02/2020
Management of colonized/infected patients for alert organisms (during hospitalization and upon discharge)	426	264 (61.97%)	162	247 (93.56%)	30/12/2009	16/12/2021	24/09/2020
Collection, storage, and transportation of laboratory samples	430	243 (56.51%)	187	223 (91.77%)	02/01/2012	31/03/2022	02/09/2020
Prevention of nosocomial pneumonia: management of oxygen therapy	430	232 (53.95%)	198	214 (92.24%)	16/03/2006	15/10/2021	05/03/2020
TB (risk assessment)	427	221 (51.76%)	206	207 (93.67%)	01/01/2005	29/03/2022	17/11/2019
Sterilization	429	198 (46.15%)	231	185 (93.43%)	01/01/2008	02/04/2022	10/02/2020
Prevention of bloodstream infections—management of central venous catheter (CVC) and peripheral venous catheter (PVC) and IV infusion setup	431	198 (45.94%)	233	184 (92.93%)	21/04/2006	25/08/2021	13/12/2019

Percentages for 'Procedure Available' are calculated relative to the total responses for that specific procedure. Percentages for 'Total Number Of Reported Revision Dates' are calculated relative to the number of available procedures. Date format is DD/MM/YYYY

### Second survey

**Fig. 1:**
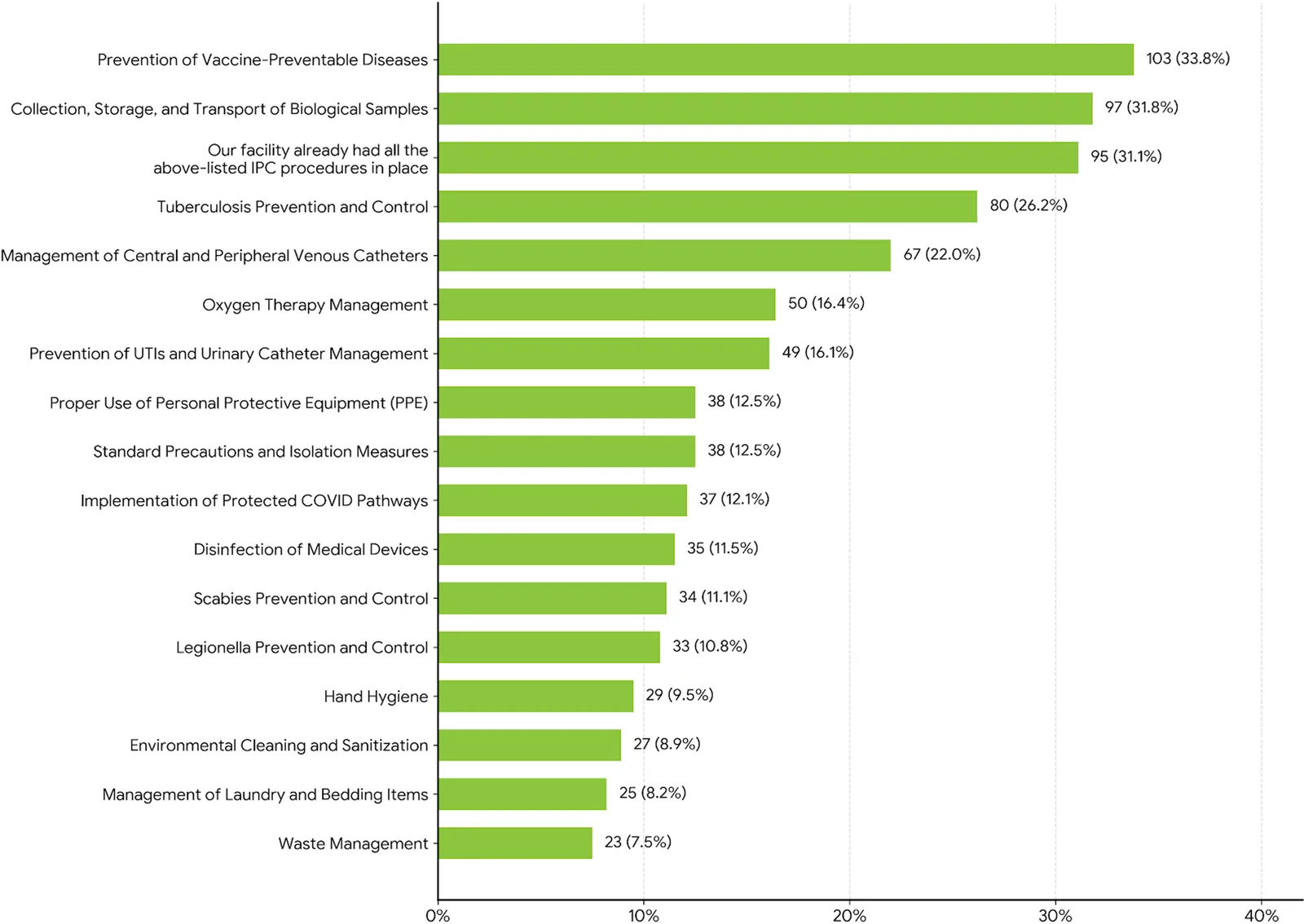
Facilities' assessment of missing and useful IPC procedures: results from the second survey (responding facilities *N* = 305). Items are sorted in descending order. Data labels indicate absolute counts and percentages relative to the total number of responding facilities

In August 2024, 305 facilities responded (39.1% of the regional census). The second survey assessed reported access/use and perceived adequacy of the standardized procedure documents; it did not assess staff adherence or clinical outcomes. Nearly all respondents judged the procedures adequate (99.7%), and 89% assigned a positive overall rating. The procedures most frequently identified as missing but useful were vaccination prophylaxis, laboratory sample management, TB control and catheter management (see Fig. 1).

## Discussion

This regional analysis documented substantial heterogeneity in the reported inclusion of IPC procedure documents in nursing-home manuals before dissemination of standardized regional materials. Lower reported inclusion was observed for several condition- or device-specific areas, including bloodstream infection prevention, sterilization, TB risk assessment, laboratory sample management and prevention of urinary tract infections. These findings are consistent with international evidence that LTCFs often face specific organizational constraints in IPC governance compared with acute-care hospitals [13].

After dissemination of the standardized procedure documents, the second survey showed high perceived adequacy and positive ratings among responding facilities. These findings suggest that centrally developed, context-adapted resources may be acceptable to nursing homes and may support harmonization of procedural documentation. However, the survey data should not be interpreted as evidence that the procedures were implemented with high fidelity or that IPC performance improved. The study assessed reported documentation and perceptions, not staff adherence, audit results, HAI rates, antimicrobial use or AMR indicators.

The distinction between procedural documentation and real-life practice is important. Written procedures are a necessary structural component of IPC programs, but they need to be supported by training, role clarity, adequate staffing, supplies, leadership commitment and monitoring. Smaller or resource-limited facilities may require additional support to adapt and implement standardized documents. Future regional work should therefore combine document governance with education, implementation support, periodic audits, vaccination and training indicators, and where feasible outcome-based surveillance such as HAI rates, antimicrobial consumption and AMR patterns [14–18].

Several limitations must be acknowledged. Both surveys relied on self-reported facility-level data, which may overestimate reported availability, updating or uptake of written procedure documents, because of social desirability and reputational concerns. Participation rates were moderate to low, and non-responders may have differed from responders in ways that could bias estimates. Facility-level characteristics, respondent professional roles and IPC infrastructure were not collected, limiting assessment of representativeness. Because survey responses were anonymous and not linked across time, we could not confirm whether the same facilities participated in both surveys or estimate facility-level change. Finally, no objective implementation or clinical outcome indicators were assessed, so causal inference about the effect of dissemination on IPC effectiveness cannot be made.

## Conclusions

The Piedmont regional initiative identified gaps and heterogeneity in IPC procedural documentation across nursing homes and developed standardized procedure documents to support harmonization. The follow-up survey documented high perceived adequacy and usefulness among respondents. These results support the value of shared regional resources as part of IPC governance in LTCFs, while underscoring that procedural documents alone do not demonstrate implementation fidelity or clinical effectiveness. Sustained training, document review, implementation support and objective monitoring indicators will be needed to assess and strengthen IPC practice in nursing homes.

## Data Availability

The datasets generated and analyzed during the current study are not publicly available because they contain facility-level organizational information collected within a regional public health initiative, but they are available from the corresponding author upon reasonable request.
